# Accuracy of Artificial Intelligence Models in Dental Implant Fixture Identification and Classification from Radiographs: A Systematic Review

**DOI:** 10.3390/diagnostics14080806

**Published:** 2024-04-11

**Authors:** Wael I. Ibraheem

**Affiliations:** Department of Preventive Dental Sciences, College of Dentistry, Jazan University, Jazan 45142, Saudi Arabia; wibraheem@jazanu.edu.sa or dr.wael007@yahoo.com

**Keywords:** artificial intelligence, deep learning, dental implant, convolutional neural network, machine learning, implant classification, implant identification, implant fixture

## Abstract

*Background and Objectives*: The availability of multiple dental implant systems makes it difficult for the treating dentist to identify and classify the implant in case of inaccessibility or loss of previous records. Artificial intelligence (AI) is reported to have a high success rate in medical image classification and is effectively used in this area. Studies have reported improved implant classification and identification accuracy when AI is used with trained dental professionals. This systematic review aims to analyze various studies discussing the accuracy of AI tools in implant identification and classification. *Methods*: The Preferred Reporting Items for Systematic Reviews and Meta-Analyses (PRISMA) guidelines were followed, and the study was registered with the International Prospective Register of Systematic Reviews (PROSPERO). The focused PICO question for the current study was “What is the accuracy (outcome) of artificial intelligence tools (Intervention) in detecting and/or classifying the type of dental implant (Participant/population) using X-ray images?” Web of Science, Scopus, MEDLINE-PubMed, and Cochrane were searched systematically to collect the relevant published literature. The search strings were based on the formulated PICO question. The article search was conducted in January 2024 using the Boolean operators and truncation. The search was limited to articles published in English in the last 15 years (January 2008 to December 2023). The quality of all the selected articles was critically analyzed using the Quality Assessment and Diagnostic Accuracy Tool (QUADAS-2). *Results*: Twenty-one articles were selected for qualitative analysis based on predetermined selection criteria. Study characteristics were tabulated in a self-designed table. Out of the 21 studies evaluated, 14 were found to be at risk of bias, with high or unclear risk in one or more domains. The remaining seven studies, however, had a low risk of bias. The overall accuracy of AI models in implant detection and identification ranged from a low of 67% to as high as 98.5%. Most included studies reported mean accuracy levels above 90%. *Conclusions*: The articles in the present review provide considerable evidence to validate that AI tools have high accuracy in identifying and classifying dental implant systems using 2-dimensional X-ray images. These outcomes are vital for clinical diagnosis and treatment planning by trained dental professionals to enhance patient treatment outcomes.

## 1. Introduction

Advancements in science and technology have influenced people’s lives in various fields, including dentistry. With the introduction of precise digital machines, dentists can provide high-quality treatment to their patients [1,2]. Various studies have shown that these computer-aided machines help dentists in various ways, from the fabrication of prostheses using CAD/CAM [2,3,4,5] to the use of robots in the treatment of patients [6,7,8]. The introduction of AI has taken dentistry to the next level. These tools help/act as supplementary aids to guide dentists’ diagnosis and treatment planning. Artificial intelligence involves developing and training machines through a set of data so that they are capable of decision making and problem solving, mimicking the human brain [9,10,11]. Machine learning (ML), a segment of AI, involves using algorithms to perform tasks without human intervention. Deep learning (DL), e.g., convolutional neural network (CNN), is an element of ML that creates a neural network capable of identifying patterns by itself, which enhances feature identification [11,12,13].

AI functions on two levels. The first level involves training, in which data are used to train and set the parameters. The second level is the testing level, in which AI performs its designated task of problem solving or decision making based on the training data. The training data are generally from the pool of collected data of interest [14,15,16,17]. Currently, AI is widely used in dentistry, which involves caries detection [18,19], periapical lesion detection [20], oral cancer diagnosis [21,22], screening of osteoporosis [23], working length determination during endodontic treatment [24,25], determination of root morphology [26,27], forensic odontology [28], pediatric dentistry [29], and implant dentistry for identification [30,31,32], diagnosis, and treatment planning [33,34]. Studies have shown that, in general, AI helps dentists in diagnosis and treatment planning, as it provides logical reasons that aid in scientific assessment.

Dental implants are commonly used for replacing missing teeth. Studies have reported a high long-term success rate with a ten-year survival rate above 95% [35,36,37,38]. With constantly increasing demands, dental implant manufacturers are developing different implant systems to increase the success rate [39]. With the increase in the use of dental implants, an increase in complications has also been reported. These complications may be related to prosthetic or fixture components or may be biological in nature [40,41,42,43]. To manage these complications, the treating dentist should know the type of implant system used so that he or she can provide the best possible treatment outcome [44]. The data related to the implant system can be retrieved easily from the patient’s previous records. However, in case of inaccessibility or loss of previous records due to any reason, it becomes difficult for the dentist to identify and classify the implant system using the available X-rays and clinical observation [45]. Dentists with vast experience in implantology may also find this task challenging. AI is reported to have a high success rate in medical image classification and is effectively used in this area. AI has been used to manage the problem of implant system identification and classification [30,31,32,46,47,48,49,50,51,52,53,54,55,56,57,58,59,60,61,62,63]. The AI tool is trained using a database of implant images and is later used to identify and classify the implants. Studies have reported improved implant classification and identification accuracy when AI is used with trained dental professionals [51,53,60,62]. This systematic review aims to analyze various studies discussing the accuracy of AI tools in implant identification and classification.

## 2. Materials and Methods

### 2.1. Registration

The Preferred Reporting Items for Systematic Reviews and Meta-Analyses (PRISMA) guidelines [64] were followed to systematize and compile this systematic review. The study was registered with the International Prospective Register of Systematic Reviews (PROSPERO registration No.: CRD42024500347).

### 2.2. Inclusion and Exclusion Criterias

The details of inclusion and exclusion criteria are given in Table 1.

### 2.3. Exposure and Outcome

In the current study, the exposure was the identification of the type and classification of an implant system using an artificial intelligence tool. The outcome was the accuracy of identification. The focused PICO (Population (P), Intervention (I), Comparison (C), and Outcome (O)) question for the current study was “What is the accuracy (outcome) of artificial intelligence tools (Intervention) in detecting and/or classifying the type of dental implant (Participant/population) using X-ray images?”

P: Human X-rays with dental implants.
I: Artificial intelligence tools.
C: Expert opinions and reference standards.
O: Accuracy of detection of the dental implant.

### 2.4. Information Sources and Search Strategy

Four electronic databases (Web of Science, Scopus, MEDLINE-PubMed, and Cochrane) were searched systematically to collect the relevant published literature. The search strings were based on the formulated PICO question. The article search was conducted in January 2024 using the Boolean operators and truncation. The search was limited to articles published in English in the last 15 years (January 2008 to December 2023). Studies performed on animals were not included. Details about the search strategy are mentioned in Table 2. Minor changes were made in the search strings based on the requirements of the database. Grey literature was searched, and bibliographies of selected studies and other review articles were checked manually to ensure that no relevant articles were left.

### 2.5. Screening, Selection of Studies, and Data Extraction

Two reviewers, M.S.A. and M.N.A., independently reviewed the titles and abstracts obtained by the electronic search. Duplicate titles were eliminated. The remaining titles were assessed based on the preset selection criteria and the PICO question. Full texts of the selected studies were reviewed independently by two reviewers, R.S.P. and W.I.I., and relevant articles were shortlisted. Any disagreements were resolved by discussion between them and with the third reviewer, M.N.A. Articles that did not meet the selection criteria were discarded, and the reason for exclusion was noted. The inter-examiner agreement was calculated using kappa statistics. W.I.I. created a data extraction chart and collected information related to the author, year of publication, country where the research was conducted, type and name of the algorithm network architecture, architecture depth, number of training epochs, learning rate, type of radiographic image, patient data collection duration, number of implant images evaluated, number and names of implant brands and models evaluated, comparator, test group, and training/validation number and ratio. Accuracy reported by the studies, author’s suggestions, and conclusions were also extracted. These data were checked and verified by a second reviewer (M.S.A.).

### 2.6. Quality Assessment of Included Studies

The quality of all the selected articles was critically analyzed using the Quality Assessment and Diagnostic Accuracy Tool (QUADAS-2) [65]. This tool is used for studies evaluating diagnostic accuracy (Table S1). This tool assesses the risk of bias and applicability concerns. The risk of bias arm has four domains that primarily focus on patient selection, index test, reference standard, and flow and timing. Meanwhile, the applicability concern arm has three domains focusing on patient selection, index test, and reference standards.

## 3. Results

### 3.1. Identification and Screening

**Figure 1:**
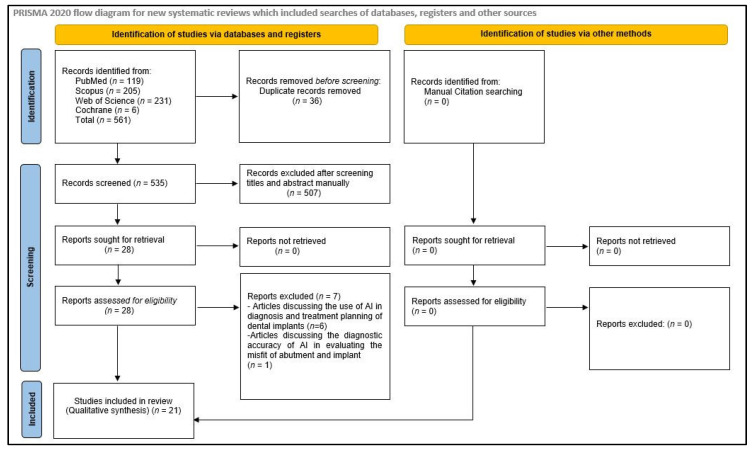
Flow chart illustrating the search strategy.

After an electronic search of the databases, 561 hits were displayed. A total of 36 articles were found to be duplicates and were removed, and the titles and abstracts of 525 articles were reviewed and checked for eligibility based on inclusion and exclusion criteria. Twenty-eight articles were selected for full-text review. Out of these twenty-eight articles, six were rejected, as they discussed the use of AI in diagnosis and treatment planning of dental implants, and one was rejected because it discussed the diagnostic accuracy of AI in evaluating the misfit of abutment and implant. Eventually, twenty-one articles were included in the study. No relevant articles meeting the selection criteria were found during the manual search of the bibliographies of the selected studies and other review articles (Figure 1). During the full-text review phase, Cohen’s kappa value was found to be 0.89 for two reviewers (R.S.P. and W.I.I.), which is an excellent agreement.

### 3.2. Study Characteristics

**Figure 2:**
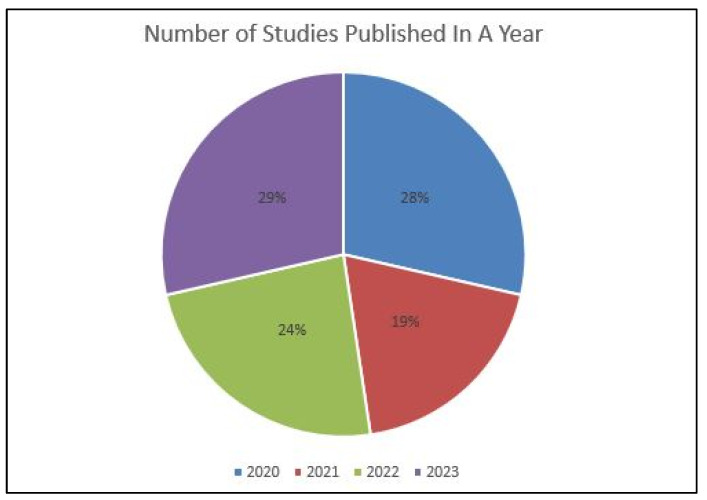
Year-wise distribution of published studies.

**Figure 3:**
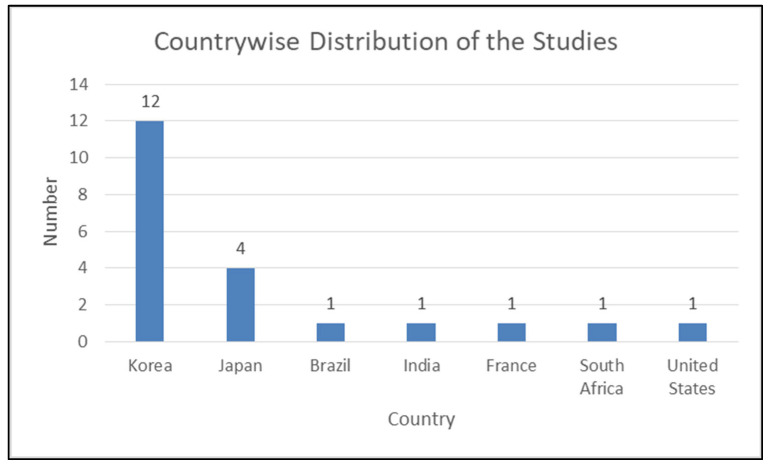
Country-wise distribution of published studies.

Table 3 displays the characteristics of studies involved in the review. All the involved studies were published in the last four years (2020: six; 2021: four; 2022: five; 2023: six) (Figure 2). Out of selected 21 studies, 12 were conducted in the Republic of Korea [31,48,51,53,54,55,57,58,59,60,61,62], four in Japan [30,47,50,52], and one each in Brazil [49], India [56], France [46], South Africa [32], and the United States [63] (Figure 3). Some of the included studies were conducted by the same research groups (Kong et al. [31,61], Park et al. [48,62], Sukegawa et al. [30,50,52], and Lee et al. [51,53,54]). Each of them shared common funding sources and grant numbers, respectively, but the studies by Kong et al. [31,61] also shared a common research registration number. The number of algorithm networks evaluated for accuracy varied in the selected studies. Ten studies [46,47,48,49,51,53,57,58,60,62] evaluated the accuracy of one algorithm network; three evaluated two algorithm networks [32,59,61]; two tested three algorithm networks [31,54]; one tested four algorithm networks [56]; three tested five algorithm networks [50,52,55]; one study each tested six [30] and ten [63] algorithm networks. All the included studies evaluated the accuracy of tested AI tools in implant detection and classification, whereas four studies [51,53,60,62] also compared this to trained dental professionals.

More than 431,000 implant images were used to train and test the selected AI tools’ implant detection and classification accuracy. Eight studies [30,31,47,50,52,56,60,61] used cropped panoramic X-ray images, and six studies [49,55,57,58,59,63] used cropped periapical X-ray images, whereas another six studies [46,48,51,53,54,62] used both periapical and panoramic implant images. In one study [32], artificially generated X-ray images were used to test AI accuracy. In most of the selected studies, the test group to training group ratio was 1:4. The learning rate of the AI algorithm ranged between 0.0001 and 0.02, the number of training epochs ranged from 50 to 2000, and the architecture depth varied from 3 to 150 layers. Also, the number of implant brands and models identified and classified varied from *N* = 3 to *N* = 130.

### 3.3. Quality Assessment of Included Studies

**Figure 4:**
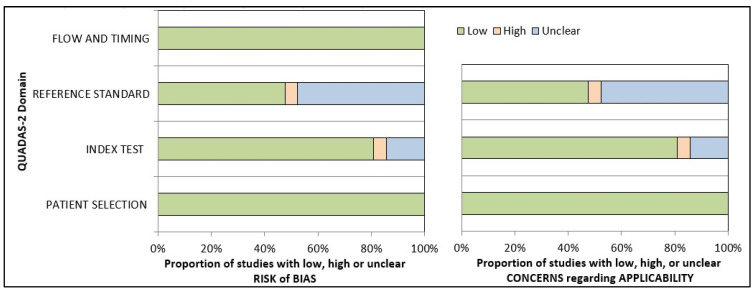
Presentation of the risk of quality assessment summary of risk bias and applicability concerns for included studies according to the QUADAS-2 tool.

The QUADAS-2 tool was used to assess the risk of bias in diagnostic tests. Out of the 21 studies evaluated, 14 were found to be at risk of bias, with high or unclear risk in one or more domains. The remaining seven studies, however, had a low risk of bias. All the included studies utilized photographic data as input to AI, resulting in a low risk of bias in the data selection domain across all studies. The results from the risk-of-bias arm demonstrated that 80.95% of the studies had a low risk, 14.28% had an unclear risk, and 4.76% had a high risk in the index test domain. In contrast, in the reference standard domain, 47.62% of the studies had a low or unclear risk of bias, while 4.76% had a high risk of bias. As the data feeding in AI technology is standardized, the final output will not affect the flow or time frame. Therefore, all studies regarded both aspects as low-risk categories (100%). Based on the risk-of-bias arm of the QUADAS-2 assessment tool, applicability concerns generated similar results. (Table S1 and Figure 4).

### 3.4. Accuracy Assessment

The overall accuracy of deep learning algorithms (DLA) in implant detection and identification ranged from a low of 67% [56] to as high as 98.5% [52]. Most included studies reported mean accuracy levels above 90% [30,46,50,51,52,53,54,55,58,59,63]. The accuracy of the latest finely tuned versions of DLAs was reported to be higher when compared to basic DLAs. Six studies [46,48,51,53,54,62] used both periapical and panoramic implant images to test the DLA models. Four studies reported higher accuracy when periapical radiographs were used [46,51,53,54,62]. One study reported higher accuracy with panoramic radiographs [48], whereas one study did not provide these details [46]. Four studies compared the accuracy of DLAs with dental professionals [51,53,60,62]. All four reported higher accuracy for DLAs when compared to dental professionals. A study by Lee et al. [60] reported that the board-certified periodontists with the assistance of DLA reported higher accuracy when compared to automated DL alone.

## 4. Discussion

The current systematic review involved all the recently published studies evaluating the accuracy of AI in implant detection and classification [30,31,32,46,47,48,49,50,51,52,53,54,55,56,57,58,59,60,61,62,63]. Overall, the outcome of this review revealed that the application of AI in implant detection and classification is a reliable and accurate method and can help dentists manage cases with no previous data related to the type of implant. With the advancements in AI, the accuracy levels may improve to a great extent.

However, the outcomes of this review should be inferred with caution because there was a significant variation between the numbers of implant models evaluated for testing the accuracy in the included studies. These ranged from as low as three [49,51,56,59] to as high as one hundred and thirty [61]. In general, the lower the number, the higher the accuracy rate of identification and classification, generally. There was a large variation in the sample size in the selected studies, which varied from 300 [57] to more than 150,000 [62].

The included studies have variations in the annotation process. PA images were used for training and testing the AI tool in six studies [49,55,57,58,59,63] and panoramic images in eight studies [30,31,47,50,52,56,60,61], whereas both PA and panoramic images were used in six studies [46,48,51,53,54,62]. One study used simulated images generated artificially [32]. In the studies where both PA and panoramic images were used, four studies reported that the accuracy of identification and classification was higher with PA images as compared to panoramic images [51,53,54,62], whereas one study reported that the accuracy was higher with the panoramic images [48].

The dental professionals involved in image selection, cropping, image standardization, training, and validation varied in areas of practice from periodontists and prosthodontists to oral and maxillofacial surgeons [30,48,51,53,54,63]. In contrast, other included studies were lacking in this information. One study validated the collected data with the help of board-certified oral and maxillofacial radiologists [48] and periodontists [53]. To reduce the heterogeneity and standardize the outcomes, the validation of the selected X-ray images should be performed by a trained radiologist. There was variation in training epochs, which varied from 50 to 2000, and the architecture depth varied from 3 to 150 layers. These parameters can affect the accuracy outcomes of the included studies. The accuracy of identification and classification also depends on the generation of Dl architecture used. There was a difference in the tested algorithms in the selected studies.

In their study, Sukegawa et al. [52] trained a CNN algorithm to analyze the implant brand and treatment stage simultaneously. The AI tool was annotated for both parameters. The classification accuracy of the implant treatment stage was reported as 0.996, with a large effect size of 0.818. The accuracy of single-task and multi-task AI tools were found to be comparable. Lee et al. [54] trained and tested the accuracy of AI tools to identify and classify fractured implants. They reported an implant classification accuracy varying from 0.804 to 0.829. They reported higher accuracy levels when DCNN architecture used only PA images for identification.

All the included studies evaluated the accuracy of tested AI tools in implant detection and classification, whereas four studies [51,53,60,62] also compared this to the trained dental professionals. Lee et al. [51,53,60] and Park et al. [62] compared the accuracy of the tested DL algorithm in implant detection and classification with trained dental professionals. All the studies reported that the accuracy performance of the DL algorithm was significantly superior when compared to humans. The accuracy reported by Park et al. [62] for DL was 82.3% and for humans varied from 16.8% (dentist not specialized in implantology) to 43.3% (dentist specialized in implantology). Lee et al. [60] reported mean accuracy of 80.56% for the automated DL algorithm, 63.13% for all participants without DL assistance, and 78.88% for all participants with DL assistance. They reported that the DL algorithm significantly helped in improving the classification accuracy of all dental professionals. Lee et al. [53], in another study, reported an accuracy of 95.4% for DL and between 50.1% to 96.8% for dentists. Another study by Lee et al. [51] reported a similar accuracy rate with DL at 97.1% and periodontists at 92.5%.

Most of the currently reported AI models use two-dimensional X-rays (periapical or panoramic). In contrast, three-dimensional X-rays like cone-beam computed tomography, widely used in implantology, were not evaluated. Also, the studies included have limitations in the type of implant systems evaluated. Thus, there is a need for more studies with a vast database that can include most of the commonly used implant systems and can utilize all forms of radiographic techniques.

The DL algorithm’s identification and classification abilities in all the selected studies were limited to the implant models the authors trained. There is a need to include more implant systems and models and create a vast database to help identify a wider variety of implant models and their characteristics. A comprehensive search strategy and rigorous selection strategy are the strong points of this systematic review. All articles mentioning AI and dental implants were assessed based on pre-set selection criteria, thus ensuring that every relevant article was reviewed.

### 4.1. Inferences and Future Directions

The field of AI is growing exponentially. There is vast literature discussing the advancements of AI in the healthcare field. Most of these AI tools focus on identification, diagnosis, and treatment planning and ways to improve them to help healthcare professionals provide the best possible treatment to their patients. All the included studies used two-dimensional images (periapical or panoramic) to identify and classify the implant systems. Three-dimensional imaging techniques like CBCT are considered a gold-standard imaging technique in dental implant planning and treatment. Thus, there is a need to develop AI tools that can use these 3D images to identify and classify the implant systems. Additionally, with the availability of newer generations of AI tools, there is a need for constant up-gradation to increase the accuracy levels of these tools.

### 4.2. Limitations

The current systematic review has a few limitations. This review included studies published only in English. The search period was limited to the last 25 years only (2008–2023). As AI is a recent and advancing field, the authors believed that conducting a search before this time may provide studies in which the technology is in an immature stage. Lastly, a meta-analysis was not feasible due to the lack of heterogeneity among the selected studies.

## 5. Conclusions

To conclude, it can be stated that the articles in the present review provide considerable evidence to validate AI tools as having high accuracy in identifying and classifying dental implant systems using 2-dimensional X-ray images. These outcomes are vital for clinical diagnosis and treatment planning by trained dental professionals to enhance patient treatment outcomes.

## Figures and Tables

**Table 1:** Selection criteria.

Inclusion Criteria	Exclusion Criteria
Literature in English language	Literature in a language other than English
Human clinical studies	Animal studies, cadaver studies, technical reports, case reports, posters, case series, reports, commentaries, reviews, unpublished abstracts and dissertations, incomplete trials, and non-peer reviewed articles
Articles published between January 2008 and December 2023	Articles published prior to January 2008
Studies evaluating the diagnostic accuracy of artificial intelligence tools in the identification and classification of dental implants	Studies evaluating the accuracy of artificial intelligence tools in identification of other dental/oral structures
Studies in which three or more implant models were identified	Studies having only the abstract and not the full text
	Studies in which less than three implant models were identified
	Studies discussing artificial intelligence tools under trial

**Table 2:** Strategy and search terms for the electronic databases.

Database	Combination of Search Terms and Strategy	Number of Titles
MEDLINE-PubMed	(((((((dental implants[MeSH Terms]) OR (dental implantation[MeSH Terms])) OR (dental implant*)) OR (dental implant system*)) OR (Dental Implant System Classification)) OR (dental implant fixture)) OR (dental implant fixture classification)) AND ((((((((((((((dental diagnostic imaging) OR (dental digital radiography[MeSH Terms])) OR (dental radiography[MeSH Terms])) OR (oral digital radiography) OR (dental Digital radiograph))) OR (Panoramic image*)) OR (panoramic radiography[MeSH Terms])) OR (Periapical images)) OR (dental radiology)) OR (periapical radiograph*)) OR (dental X-ray image)) OR (synthetic dental X-ray image)) OR (OPG)) OR (Orthopantomogram)) OR (Intro oral radiograph) AND ((((((((((((((((artificial intelligence[MeSH Terms]) OR (machine learning[MeSH Terms])) OR (neural networks computer[MeSH Terms])) OR (algorithms[MeSH Terms])) OR (deep learning)) OR (supervised machine learning)) OR (Automated deep learning)) OR (Object detection)) OR (Yolov3)) OR (object detection algorithm)) OR (convolutional neural network*)) OR (Deep Neural Network*)) OR (multi-task learning)) OR (deep convolutional neural network)) OR (Transfer Learning)) OR (attention branch network)) OR (Ensemble Deep Learning) AND (((((((((sensitivity and specificity[MeSH Terms]) OR (Accuracy)) OR (sensitivity)) OR (specificity)) OR (Positive Predictive Value*)) OR (Negative Predictive Value*)) OR (Precision)) OR (Recall)) OR (F1 score)) OR (Area under receiver operating characteristics curve) Filters: Humans, English, from 1 January 2008 to 31 December 2023	119
Scopus	(“dental implants” OR “dental implantation” OR “dental implant*” OR “dental implant system*” OR “Dental Implant System Classification” OR “dental implant fixture” OR “dental implant fixture classification”) AND (“dental diagnostic imaging” OR “dental digital radiography” OR “dental radiography” OR “oral digital radiography” OR “dental Digital radiograph” OR “Panoramic image*” OR “panoramic radiography” OR “Periapical images” OR “dental radiology” OR “periapical radiograph*” OR “dental X-ray image” OR “synthetic dental X-ray image” OR “OPG” OR “Orthopantomogram” OR “Intro oral radiograph”) AND (“artificial intelligence” OR “machine learning” OR “neural networks computer” OR “algorithms” OR “deep learning” OR “supervised machine learning” OR “Automated deep learning” OR “Object detection” OR “Yolov3” OR “object detection algorithm” OR “convolutional neural network*” OR “Deep Neural Network*” OR “multi-task learning” OR “deep convolutional neural network” OR “Transfer Learning” OR “attention branch network” OR “Ensemble Deep Learning”) AND (“sensitivity and specificity” OR “Accuracy” OR “sensitivity” OR “specificity” OR “Positive Predictive Value*” OR “Negative Predictive Value*” OR “Precision” OR “Recall” OR “F1 score” OR “Area under receiver operating characteristics curve”) AND PUBYEAR > 2008 AND PUBYEAR <2023 AND (LIMIT-TO (SUBJAREA, “DENT”)) AND (LIMIT-TO (DOCTYPE, “ar”)) AND (LIMIT-TO (LANGUAGE, “English”)) AND (LIMIT-TO(SRCTYPE, “j”))	205
Web of Science	#1 (P) TS = (‘dental implants’ OR ‘dental implantation’ OR ‘dental implant*’OR ‘dental implant system*’ OR ‘Dental Implant System Classification’ OR ‘dental implant fixture’ OR ‘dental implant fixture classification’ OR ‘dental diagnostic imaging’ OR ‘dental digital radiography’ OR ‘dental radiography’ OR ‘oral digital radiography’ OR ‘dental Digital radiograph’ OR ‘Panoramic image*’ OR ‘panoramic radiography’ OR ‘Periapical images’ OR ‘dental radiology’ OR ‘periapical radiograph*’ OR ‘dental X-ray image’ OR ‘synthetic dental X-ray image’ OR ‘OPG’ OR Orthopantomogram OR ‘Intro oral radiograph’) #2 (I) TS = (‘artificial intelligence’ OR ‘machine learning’ OR ‘neural networks computer’ OR algorithms OR ‘deep learning’ OR ‘supervised machine learning’ OR ‘Automated deep learning’ OR ‘Object detection’ OR ‘Yolov3′ OR ‘object detection algorithm’ OR ‘convolutional neural network*’ OR ‘Deep Neural Network*’ OR ‘multi-task learning’ OR ‘deep convolutional neural network’ OR ‘Transfer Learning’ OR ‘attention branch network’ OR ‘Ensemble Deep Learning’) #3 (O) TS = (‘sensitivity and specificity’ OR Accuracy OR sensitivity OR specificity OR ‘Positive Predictive Value*’ OR ‘Negative Predictive Value*’ OR Precision OR Recall OR ‘F1 score’ OR ‘Area under receiver operating characteristics curve’) #3 AND #2 AND #1 Indexes = SCI-EXPANDED, SSCI, A&HCI, CPCI-S, CPCI-SSH, ESCI, CCR-EXPANDED, IC Timespan = January 2008 to December 2023 and English (Languages)	8
Cochrane Library	#1 MeSH descriptor: [Dental Implants] explode all trees #2 MeSH descriptor: [Dental Implantation] explode all trees #3 dental implant* #4 dental implant system* #5 Dental Implant System Classification #6 dental implant fixture #7 dental implant fixture classification #8 dental diagnostic imaging #9 MeSH descriptor: [Radiography, Dental, Digital] explode all trees #10 MeSH descriptor: [Radiography, Dental] explode all trees #11 oral digital radiography #12 dental Digital radiograph #13 Panoramic image* #14 panoramic radiography #15 Periapical images #16 dental radiology #17 periapical radiograph* #18 dental X-ray image #19 synthetic dental X-ray image #20 OPG #21 Orthopantomogram #22 Intro oral radiograph #23 MeSH descriptor: [Artificial Intelligence] explode all trees #24 MeSH descriptor: [Machine Learning] explode all trees #25 MeSH descriptor: [Neural Networks, Computer] explode all trees #26 MeSH descriptor: [Algorithms] explode all trees #27 deep learning #28 supervised machine learning #29 Automated deep learning #30 Object detection #31 Yolov3 #32 object detection algorithm #33 convolutional neural network* #34 Deep Neural Network* #35 multi-task learning #36 deep convolutional neural network #37 Transfer Learning #38 attention branch network #39 Ensemble Deep Learning #40 MeSH descriptor: [Sensitivity and Specificity] explode all trees #41 Accuracy #42 sensitivity #43 specificity #44 Positive Predictive Value* #45 Negative Predictive Value* #46 Precision #47 Recall #48 F1 score #49 Area under receiver operating characteristics curve #50 #1 OR #2 OR #3 OR #4 OR #5 OR #6 OR #7 #51 #8 OR #9 OR #10 OR #11 OR #12 OR #13 OR #14 OR #15 OR #16 OR #17 OR #18 OR #19 OR #20 OR #21 OR #22 #52 #23 OR #24 OR #25 OR #26 OR #27 OR #28 OR #29 OR #30 OR #31 OR #32 OR #33 OR #34 OR #35 OR #36 OR #37 OR #38 OR #39 #53 #40 OR #41 OR #42 OR #43 OR #44 OR #45 OR #46 OR #47 OR #48 OR #49 #54 #50 AND #51 AND #52 AND #53	6

* truncation. P, population; I, intervention; O, outcome.

**Table 3:** Study characteristics and accuracy results of the included studies.

Author, Year Country	Algorithm Network Architecture and Name	Architecture Depth (Number of Layers), Number of Training Epochs, and Learning Rate	Type of Radiographic Image	Patient Data Collection/X-ray Collection Duration	Number of X-rays/Implant Images Evaluated *(N)*	Number and Names of Implant Brands and Models Evaluated	Comparator	Test Group and Training/Validation Number and Ratio	Accuracy Reported	Authors Suggestions/Conclusions
Kong et al., 2023, Republic of Korea [61]	- 2 DL - YOLOv5 - YOLOv7	-Training Epochs: YOLOv5: 146, 184, 200 YOLOv7: 200	Pano	2001 to 2021	*N* = 14,037	Implant models: *N* = 130 * Implant design classification: 1. Coronal one-third 2. Middle one-third 3. Apical part	EORS	Test Group: 20% Training Group: 80%	mAP (area under the precision–recall curve): YOLOv5: Implant-dataset-1:0.929 Implant-dataset-2: 0.940 Implant-dataset-3:0.873 YOLOv7: Implant-dataset-1: 0.931 Implant-dataset-2: 0.984 Implant-dataset-3: 0.884 YOLOv7 Implant-dataset-1: IPA: 0.986 IPA + Magnification ×2: 0.988 IPA + Magnification ×4: 0.986	mAP: YOLOv7 > YOLOv5 The tested DL has a high accuracy
Kong, 2023, Republic of Korea [58]	- Fine-tuned CNN - Google automated machine learning (AutoML) Vision	Training: 32 node hours	PA	January 2005 to December 2019	*N* = 4800	Implant Brands: *N* = 3 (A) Osstem Implant (B) Biomet 3i LLC (C) Dentsply Sirona Implant models: *N* = 4 (1) Osstem TSIII (25%) (2) Osstem USII (25%) (3) Biomet 3i Osseotite External (25%) (4) Dentsply Sirona Xive (25%)	EORS	Test Group: 10% Training Group: 80% Fine-tuning Group:10%	Overall Accuracy: 0.981 Precision: 0.963 Recall: 0.961 Specificity: 0.985 F1 score: 0.962	Tested fine-tuned CNN showed high accuracy in the classification of DISs
Park et al., 2023, Republic of Korea [62]	- Fine-tuned and pretrained DL - ResNet-50	- Depth: 50 layers	PA and Pano	NM	*N* = 150,733 PA (24.8%) and Pano (75.2%)	Implant Brands: *N* = 10 (A) Neobiotech (*n* = 14.1%) (B) NB (*n* = 2.41%) (C) Dentsply (*n* = 10.14%) (D) Dentium (*n* = 27.26%) (E) Dio (*n* = 1.01%) (F) Megagen (*n* = 5.17%) (G) ST (*n* = 3.30%) (H) Shinhung (*n* = 2.23%) (I) Osstem (*n* = 28.47%) (J) Warantec (*n* = 5.86%) Implant Models: *N* = 25 (A) Neobiotech: (1) IS I 1, (2) IS II, (3) IS III, (4) EB; (B) NB: (1) Branemark; (C) Dentsply: (1) Astra, (2) Xive; (D) Dentium: (1) Implantium, (2) Superline; (E) Dio: (1) UF, (2) UF II; (F) Megagen: (1) Any ridge, (2) Anyone internal, (3) Anyone external, (4) Exfeel external; (G) ST: (1) TS standard, (2) TS standard plus, (3) Bone level; (HI) Shinhung: (1) Luna; (I) Osstem: (1) GS II, (2) SS II, (3) TS III, (4) US II, (5) US III; (J) Warantec: (1) Hexplant	DL vs. 28 dental professionals (9 dentists specialized in implantology and 19 dentists not specialized in implantology)	Training Group: 80% Validation Group: 10% Test Group: 10%	Accuracy (1) DL: (a) Both Pano and PA: 82.3% (95% CI, 78.0–85.9%) (b) PA: 83.8% (95% CI, 79.6–87.2%) (c) Pano: 73.3% (95% CI, 68.5–77.6%) (2) All dental professionals: (a) Both Pano and PA: 23.5% ± 18.5 (b) PA: 26.2% ± 18.2 (c) Pano: 24.5% ± 19.0 (3) Dentist specialized in Implantology: (a) Both Pano and PA: 43.3% ± 20.4 (b) PA: 43.3% ± 19.7 (c) Pano: 43.2% ± 21.2 (4) Dentist not specialized in Implantology: (a) Both Pano and PA: 16.8% ± 9 (b) PA: 18.1% ± 9.9 (c) Pano: 15.6% ± 8.5 Deep learning (For both Pano and PA) AUC: 0.823 Sensitivity: 80.0% Specificity: 84.5% PPV: 83.8% NPV: 80.9%	Classification accuracy performance of DL was significantly superior
Hsiao et al., 2023, USA [63]	- 10 CNN architectures (1) MnasNet (2) ShuffleNet7 (3) MobileNet8 (4) AlexNet9 (5) VGG10 (6) ResNet11 (7) DenseNet12 (8) SqueezeNet13 (9) ResNeXt14 (10) Wide ResNet15	- Learning rate: 0.001 - For training accuracy, the CNN assessed data 90 times per image	PA	January 2011 to January 2019	*N* = 788	Implant Brands: *N* = 3 (A) BioHorizons (22.84%) (B) ST (34.51%) (C) NB (42.63%) Implant Models (A) BioHorizons: (1) Legacy implant Tapered Pro; (B) ST: (1) Bone Level, Bone Level Tapered, Standard Straumann, Tapered Effect; (C) NB: (1) Active, (2) Parallel, (3) Replace, (4) Replace Select Straight, (5) Replace Select Tapered, (6) Speedy Groovy, (7) Speedy Replace	EORS	Training Group: 75% Test Group: 25%	Overall implant-identification Accuracy: >90% Test accuracy (1) MnasNet6: 81.89% (2) ShuffleNet7: 96.85% (3) MobileNet8: 92.68% (4) AlexNet9: 94.35% (5) VGG10: 92.94% (6) ResNet11: 96.43% (7) DenseNet12: 96.41% (8) SqueezeNet13: 91.55% (9) ResNeXt14: 93.90% (10) Wide ResNet15: 92.01%	Tested CNN has high accuracy and speed
Park et al., 2023, Republic of Korea [48]	- Customized automatic DL - Neuro-T version 3.0.1	- Training epochs: 500	PA and Pano	NM	*N* = 156,965 (Pano: 116,756; PA: 40,209)	Implant Brands: *N* = 10 (A) Neobiotech, (B) NB, (C) Dentsply, (D) Dentium, (E) Dioimplant, (F) Megagen, (G) ST, (H) Shinhung, (I) Osstem, (J) Warantec Implant models: *N* = 27 1. IS I (Neobiotech) (5%); 2. IS II (Neobiotech) (1.83%); 3. IS III (Neobiotech) (5.18%); 4. EB (Neobiotech) (1.53%); 5. Branemark (NB) (2.32%); 6. Astra (Dentsply) (8.90%); 7. Xive (Dentsply) (0.84%); 8. Implatinum (Dentium) (12.20%); 9. Superline (Dentium) (13.98%); 10. UF (Dioimplant) (0.51%); 11. UF II (Dioimplant (0.47%); 12. Any ridge (Megagen) (0.22%); 13. Anyone international (Megagen) (2.43%); 14. Anyone external (Megagen) (1.63%); 15. Exfeel external (Megagen) (0.69%); 16. TS standard (Straumann) (0.85%); 17. TS standard plus (Straumann) (0.66%); 18. Bone level (Straumann) (1.66%); 19. Luna (Shinhung) (2.15%); 20. GS II (Osstem) (1.10%); 21. SS II (Osstem) (0.53%); 22. TS III (Osstem) (18.96%); 23. US II (Osstem) (6.15%); 24. US III (Osstem) (0.60%); 25. Hexplant (Warantec) (5.63%); 26. Internal (Warantec) (3.68%); 27. IT (Warantec) (0.28%)	EORS	Test Group: 10% Training Group: 80% Validation Group: 10%	Overall 1. Accuracy: 88.53% 2. Precision: 85.70% 3. Recall: 82.30% 4. F1 score: 84.00% Using Pano: 1. Accuracy: 87.89% 2. Precision: 85.20% 3. Recall: 81.10% 4. F1 score: 83.10% Using PA: 1. Accuracy: 86.87% 2. Precision: 84.40% 3. Recall: 81.70% 4. F1 score: 83.00%	- DL has reliable classification accuracy - No statistically significant difference in accuracy performance between the Pano and PA - Suggestion: Additional dataset needed for confirming clinical feasibility of DL
Kong et al., 2023, Republic of Korea [31]	3 DLs (1) EfficientNet (2) Res2Next (3) Ensemble model	NM	Pano	March 2001 and April 2021	*N* = 45,909	Implant Brands: *N* = 20 (A) Bicon; (B) BioHorizons; (C) BIOMET 3i; (D) Biotem; (E) Dental Ratio; (F) Dentis; (G) Dentium; (H) Dentsply Sirona; (I) Dio Implant; (J) Hi ossen Implant; (K) IBS Implant; (L) Keystone Dental; (M) MegaGen Implant; (N) Neobiotech; (O) NB; (P) Osstem Implant; (Q) Point Implant; (R) ST; (S) Thommen Medical; (T) Zimmer Dental Implant models: *N* = 130 *	EORS	Test Group:20% Training Group: 80%	Top-1 accuracy (ratio that the nearest class was predicted, and the answer was correct) (a) EfficientNet: 73.83 (b) Res2Next: 73.09 (c) Ensemble model: 75.27 Top-5 accuracy (ratio in which the five nearest classes were predicted, and the answer was among them) (a) EfficientNet: 93.84 (b) Res2Next: 93.60 (c) Ensemble model: 95.02 Precision: (a) EfficientNet: 74.61 (b) Res2Next: 77.79 (c) Ensemble model: 78.84 Recall: (a) EfficientNet: 73.83 (b) Res2Next: 73.08 (c) Ensemble model: 75.27 F1 score: (a) EfficientNet: 72.02 (b) Res2Next: 73.55 (c) Ensemble model: 74.89	Accuracy: Ensemble model > EfficientNet > Res2Next
Jang et al., 2022, Republic of Korea [57]	Faster R-CNN Resnet 101	- Training epochs: 1000	PA	January 2016 to June 2020	*N* = 300	NM	EORS	Test Group: 20% Training Group: 80%	Classification: Precision: 0.977 Recall: 0.992 F1 score: 0.984	Faster R-CNN model provided high-quality object detection for dental implants and peri-implant tissues
Kohlakala et al., 2022, South Africa [32]	DL (1) FCN-1 (2) FCN-2	- Training epochs: 1000	Artificially generated (simulated) X-ray images	NM	NM	Implant brands: *N* = 1 MIS Dental implant Implant models: *N* = 9 (1) Conical narrow platform V3 (2) Conical narrow platform C1 (3) Conical standard platform V3 (4) Conical wide platform C1 Internal diameter 4.00 mm (5) Internal hex narrow platform SEVEN (6) Internal hex standard platform SEVEN (7) Internal hex wide platform SEVEN (8) External hex standard platform LANCE (9) External hex wide platform	EORS	Test Group: 17–18% Training Group: 82–83% Validation: 12–13%	Semi-automated system (human jaws) Full precision: 70.52% Recall: 69.76% Accuracy: 69.76% F1 score: 69.70% Fully automated system (human jaws) Full precision: 70.55% Recall: 68.67% Accuracy: 68.67% F1 score: 67.60%	Proposed fully automated system displayed promising results for implant classification
Sukegawa et al., 2022, Japan [30]	CNN and CNN + ABN (1) ResNet18 (2) ResNet18 + ABN (3) ResNet50 (4) ResNet50 + ABN (5) ResNet152 (6) ResNet152 + ABN	- Depth: (1 and 2) 18 layers (3 and 4) 50 layers (5 and 6) 152 layers - Training epochs: 100 - Learning rate: 0.001	Pano	NM	*N* = 10,191	Implant brands: *N* = 5 (A) ZB (4.19%) (B) NB (25.21%) (C) Kyocera Co. (7.07%) (D) ST (8.94%) (E) Dentsply IH AB (54.16%) Implant models: *N* = 13 1. Full OSSEOTITE 4.0 (4.19%) 2. Astra EV 4.2 (8.29%) 3. Astra TX 4.0 (24.73%) 4. Astra TX 4.5 (10.93%) 5. Astra Micro Thread 4.0 (6.91%) 6. Astra Micro Thread 4.5 (3.73%) 7. Branemark Mk III 4.0 (3.48%) 8. FINESIA 4.2 (3.33%) 9. POI EX 42 (3.74%) 10. Replace Select Tapered 4.3 (6.04%) 11. Nobel Replace CC 4.3 (15.69%) 12. Straumann Tissue 4.1 (6.43%) 13. Straumann Bone Level 4.1 (2.51%)	EORS	Test dataset split Training: validation: 8:2.	Test accuracy (95% CI): (a) ResNet18: 0.9486 (b) ResNet18 + ABN: 0.9719 (c) ResNet50: 0.9578 (d) ResNet50 + ABN: 0.9511 (e) ResNet152: 0.9624 (f) ResNet152 + ABN: 0.9564 Precision: (a) ResNet18: 0.9441 (b) ResNet18 + ABN: 0.9686 (c) ResNet50: 0.9546 (d) ResNet50 + ABN: 0.9477 (e) ResNet152: 0.9575 (f) ResNet152 + ABN: 0.9514 Recall: (a) ResNet18: 0.9333 (b) ResNet18 + ABN: 0.9627 (c) ResNet50: 0.9471 (d) ResNet50 + ABN: 0.9382 (e) ResNet152: 0.9509 (f) ResNet152 + ABN: 0.9450 F1 score: (a) ResNet18: 0.9382 (b) ResNet18 + ABN: 0.9652 (c) ResNet50: 0.9498 (d) ResNet50 + ABN: 0.9416 (e) ResNet152: 0.9530 (f) ResNet152 + ABN: 0.9470 AUC: (a) ResNet18: 0.9979 (b) ResNet18 + ABN: 0.9993 (c) ResNet50: 0.9983 (d) ResNet50 + ABN: 0.9975 (e) ResNet152: 0.9985 (f) ResNet152 + ABN: 0.9955	ResNet 18 showed very high compatibility in the ABN model Accuracy: ResNet18 + ABN > ResNet152 > ResNet50: 0.9578 > ResNet152 + ABN > ResNet50 + ABN > ResNet18
Kim et al., 2022, Republic of Korea [59]	- DCNN - YOLOv3 (Darknet-53)	- Depth: 53 layers - Training epochs: 100, 200, and 300	PA	April 2020 to July 2021	*N* = 355	Implant models: *N* = 3 (1) Superline (Dentium Co. Ltd., Seoul, Republic of Korea) (34.08%) (2) TS III (Osstem Implant Co. Ltd., Seoul, Republic of Korea) (32.39%) (3) Bone Level Implant (Institut ST AG, Basel, Switzerland) (33.52%)	EORS	Test Group: 20% Training Group: 80% (10% used for validation)	At 200 epochs training: Accuracy: 96.7% Sensitivity: 94.4% Specificity: 97.9% Confidence score: 0.75	High performance could be achieved with YOLOv3 DCNN
Lee et al., 2022, Republic of Korea [60]	- Automated DL - Neuro-T version 2.0.1, Neurocle Inc., Seoul, Republic of Korea	NM	Pano	NM	*N* = 180	Implant models: *N* = 6 (1) Astra OsseoSpeed^®^ TX (16.66%) (2) Dentium Implantium^®^ (16.66%) (3) Dentium Superline^®^ (16.66%) (4) Osstem TSIII^®^ (16.66%) (5) Straumann SLActive^®^ BL (16.66%) (6) Straumann SLActive^®^BLT (16.66%).	DL vs. 44 dental professionals (5 board-certified periodontists, 8 periodontology residents, 17 conservative and pediatric dentistry residents, and 14 interns)	Training Group: 80% Validation Group: 20%	Mean Accuracy - Automated DL algorithm: 80.56% - All participants (without DL assistance): 63.13% -- All participants (with DL assistance): 78.88%	The DL algorithm significantly helps improve the classification accuracy of dental professionals Average accuracy: board-certified periodontists with DL > Automated DL
Benakatti et al., 2021, India [56]	4 machine learning algorithms: (1) Support vector machine (SVM) (2) Logistic regression (3) K-nearest neighbor (KNN) (4) X boost classifiers	NM	Pano	January 2021 to April 2021	NM	Implant models: *N* = 3 1. Osstem TS III SA Regular, 2. Osstem TS III SA Medium, 3. Noris Medical Tuff.	EORS	Test Group: 20% Training Group: 80%	Average accuracy overall: 0.67 Accuracy based on Hu moments (a) SVM:0.47 (b) Logistic regression: 0.33 (c) KNN: 0.50 (d) X boost classifiers: 0.33 Accuracy based on eigenvalues (a) SVM: 0.67 (b) Logistic regression: 0.17 (c) KNN: 0.67 (d) X boost classifiers: 0.67	The machine learning models tested are proficient enough to identify DISs Accuracy: logistic regression > SVM > KNN > X boost
Santos et al., 2021, Brazil [49]	- DCNN - Stochastic Gradient Descent optimization algorithm	- Depth: 5 convolutional layers + 5 dense layers - Training epochs: 25 - Learning rate: 0.005	PA	2018–2020	*N* = 1800	Implant Brands and Model: *N* = 3 (A) ST (internal-connection) (33.33%) (B) Neodent (Neodent) (33.33%) (C) SIN Implante (SIN Morse taper with prosthetic platform) (33.33%)	EORS	Test Group: 20% Training Group: 80%	1. Accuracy = 85.29% (78.4% to 90.5%) 2. Sensitivity = 89.9% (81.1% to 95.6%) 3. Specificity = 82.4% (73.7% to 87.3%) 4. PPV = 82.6% (74.1% to 86.6%) 5. NPV = 88.5% (79.8% to 93.9%	- DCNN has high degree of accuracy for implant identification - Suggestion: Need for more comprehensive database
Sukegawa et al., 2021, Japan [52]	5 CNNs 1. ResNet18 2. ResNet34 3. ResNet50 4. ResNet101 5. ResNet152	- Depth: 1. 18 layers 2. 34 layers 3. 50 layers 4. 101 layers 5. 152 layers - Training epochs: 50 - Learning rate: 0.001	Pano	January 2005 to December 2020	*N* = 9767	Implant brands: *N* = 5 (A) ZB, (B) Dentsply, (C) NB, (D) Kyocera, (E) ST Implant models: *N* = 12 1. Full OSSEOTITE 4.0 (ZB) (4.37%); 2. Astra EV 4.2 (Dentsply) (8.65%); 3. Astra TX 4.0 (Dentsply) (25.80%); 4. Astra MicroThread 4.0 (Dentsply) (7.20%); 5. Astra MicroThread 4.5 (Dentsply) (3.89%); 6. Astra TX 4.5 (Dentsply) (11.40%); 7. Brånemark Mk III 4.0 (NB) (3.63%); 8. FINESIA 4.2 (Kyocera) (3.39%); 9. Replace Select Tapered 4.3 (NB) (6.30%); 10. Nobel CC 4.3 (NB) (16.37%); 11. Straumann Tissue 4.1 (ST) (6.70%); 12. Straumann Bone Level 4.1 (ST) (2.25%)	EORS	Validation Group: 20% Training Group: 80%	Single task: accuracy: (a) ResNet18: 0.9787 (b) ResNet34: 0.9800 (c) ResNet50: 0.9800 (d) ResNet101: 0.9841 (e) ResNet152: 0.9851 Precision: (a) ResNet18: 0.9737 (b) ResNet34: 0.9790 (c) ResNet50: 0.9816 (d) ResNet101: 0.9822 (e) ResNet152: 0.9839 Recall: (a) ResNet18: 0.9726 (b) ResNet34: 0.9743 (c) ResNet50: 0.9746 (d) ResNet101: 0.9789 (e) ResNet152: 0.9809 F1 score: (a) ResNet18: 0.9724 (b) ResNet34: 0.9762 (c) ResNet50: 0.9776 (d) ResNet101: 0.9805 (e) ResNet152: 0.9820 AUC: (a) ResNet18: 0.9996 (b) ResNet34: 0.9997 (c) ResNet50: 0.9996 (d) ResNet101: 0.9997 (e) ResNet152: 0.9998	- CNNs conferred high validity in the classification of DISs - The larger the number of parameters and the deeper the network, the better the performance for classifications
Lee et al., 2021, Republic of Korea [54]	- 3 DCNN (1) VGGNet-19 (2) GoogLeNet Inception-v3 (3) Automated DCNN (Neuro-T version 2.1.1)	- Depth: (1) 19 layers (2) 22 layers (3) 18 layers - Training epochs: 2000 - Learning rate: 0.0001	PA and Pano	January 2006 to December 2019	251 intact and 198 fractured dental implants images (Pano: 45.2%, PA: 54.8%)	Not mentioned Intact and fractured dental implants were identified and classified	EORS	Test Group: 20% Training Group: 60% Validation Group: 20%	Overall: AUC: - VGGNet-19: 0.929 (95% CI: 0.854–0.972) - GoogLeNet Inception-v3: 0.967 (95% CI: 0.906–0.993) - Automated DCNN: 0.972 (95% CI: 0.913–0.995) Sensitivity: - VGGNet-19: 0.933 - GoogLeNet Inception-v3: 1.00 -Automated DCNN: 0.866 Specificity: - VGGNet-19: 0.933 - GoogLeNet Inception-v3: 0.866 -Automated DCNN: 0.966 Youden index: - VGGNet-19: 0.866 - GoogLeNet Inception-v3: 0.866 - Automated DCNN: 0.833	- All tested DCNNs showed acceptable accuracy in the detection and classification of fractured dental implants - Best accuracy: Automated DCNN architecture using only PA images
Hadj Saïd et al., 2020, France [46]	- DCNN - Pretrained GoogLeNet Inception v3	- Depth: 22 layers deep (27 including the pooling layers) - Training epochs: 1000 - Learning rate: 0.02	PA and Pano	NM	*N* = 1206	Implant brands: *N* = 3 (A) NB (49.4%), (B) ST (25.5%), (C) ZB (25%) Implant models: *N* = 6 1. NobelActive (21.64%); 2. Brånemark system (21.77%); 3. Straumann Bone Level (12.43%); 4. Straumann Tissue Level (13.1%); 5. Zimmer Biomet Dental Tapered Screw-Vent (12.6%); 6. SwissPlus (Zimmer) (12.43%)	EORS	Test Group: 19.9% Training and Validation Group: 80%	1. Diagnostic accuracy = 93.8% (87.2% to 99.4%) 2. Sensitivity = 93.5% (84.2% to 99.3%) 3. Specificity = 94.2% (83.5% to 99.4%) 4. PPV = 92% (83.9% to 97.2%) 5. NPV = 91.5% (80.2% to 97.1%)	- Good performance of DCNN in implant identification - Suggestion: Creation of a giant database of implant radiographs
Lee et al., 2020, Republic of Korea [53]	- Automated DCNN - Neuro-T version 2.0.1 (Neurocle Inc., Republic of Korea)	- Depth: 18 layers	PA and Pano	January 2006 to May 2019	*N* = 11,980 (Pano: 59.6% and PA: 40.4%)	Implant brands: *N* = 4 (A) Osstem implant system (46.9%) (B) Dentium implant system (40.7%) (C) Institut ST implant system (9.2%) (D) Dentsply implant system (3.2%) Implant models: *N* = 6 1. Astra OsseoSpeed TX (Dentsply) (3.2%) 2. Implantium (Dentium) (21%) 3. Superline (Dentium) (19.7%) 4. TSIII^®^ (Osstem) (46.9%) 5. SLActive BL (Institut ST) (4.5%) 6. SLActive BLT (Institut ST) (4.7%)	DCNN vs. 25 dental professionals (board-certified periodontist, periodontology residents, other specialty residents)	Test Group: 20% Training Group: 80%	DCNN overall (based on 180 Images): Accuracy (AUC): 0.954 Youden index: 0.808 Sensitivity: 0.955 Specificity: 0.853 Using Pano images: AUC: 0.929 Youden index: 0.804 Sensitivity: 0.922 Specificity: 0.882 Using PA images: AUC: 0.961 Youden index: 0.802 Sensitivity: 0.955 Specificity: 0.846 AUC Dental Professionals: i. Board-certified periodontist: 0.501–0.968 ii. Periodontology residents: 0.503–0.915 iii. Other specialty residents: 0.544–0.915	Accuracy: DCNN > Dental professionals
Takahashi et al., 2020, Japan [47]	- DL - Fine-tuned Yolo v3	- Training epochs: 1000 - Learning rate: 0.01	Pano	Feb. 2000–2020	*N* = 1282	Implant brands: *N* = 3 (A) NB, (B) ST, (C) GC Implant models: *N* = 6 1. MK III (NB); 2. MK III Groovy (NB); 3. Bone level implant (ST); 4. Genesio Plus ST (Genesio) (GC); 5. MK IV (NB); 6. Speedy Groovy (NB)	EORS	Test Group: 20% Training Group: 80%	1. True-positive ratio: 0.50 to 0.82 2. Average precision: 0.51 to 0.85 3. Mean average precision: 0.71 4. Mean intersection over union: 0.72	- Implants can be identified by using DL - Suggestion: More images of other implant systems will be necessary to increase the learning performance
Sukegawa et al., 2020, Japan [50]	- 5 DCNNs 1. Basic CNN 2. VGG16 transfer-learning model 3. Finely tuned VGG16 4. VGG19 transfer-learning model 5. Finely tuned VGG19	- Depth: 1: 3 convolution layers, 2 and 3: 16 layers (13 convolutional layers + 3 fully connected layers), 4 and 5: 19 layers (16 convolutional layers + 3 fully connected layers) - Training epochs: 700 - Learning rate: 0.0001	Pano	January 2005 to December 2019	*N* = 8859	Implant brands: *N* = 5 (A) ZB, (B) Dentsply, (C) NB, (D) Kyocera, (E) ST Implant models: *N* = 11 1. Full OSSEOTITE 4.0 (ZB) (4.81%); 2. Astra EV 4.2 (Dentsply) (4.79%); 3. Astra TX 4.0 (Dentsply) (28.45%); 4. Astra MicroThread 4.0 (Dentsply) (12.28%); 5. Astra MicroThread 4.5 (Dentsply) (7.87%); 6. Astra TX 4.5 (Dentsply) (4.36%); 7. Brånemark Mk III 4.0 (NB) (4.77%); 8. FINESIA 4.2 (Kyocera) (2.63%); 9. Replace Select Tapered 4.3 (NB) (5.48%); 10. Nobel CC 4.3 (NB) (18.97%); 11. Straumann Tissue 4.1 (ST) (5.53%)	EORS	Test Group: 25% Training Group: 75%	1. Basic CNN: i. Accuracy: 0.860 ii. Precision: 0.842 iii. Recall: 0.802 iv. F1 score: 0.819 2. VGG16-transfer learning: i. Accuracy: 0.899 ii. Precision: 0.888 iii. Recall: 0.864 iv. F1 score: 0.874 3. Finely tuned VGG16: i. Accuracy: 0.935 ii. Precision: 0.928 iii. Recall: 0.907 iv. F1 score: 0.916 4. VGG19 transfer-learning: i. Accuracy: 0.880 ii. Precision: 0.873 iii. Recall: 0.840 iv. F1 score: 0.853 5. Finely tuned VGG19: i. Accuracy: 0.927 ii. Precision: 0.913 iii. Recall: 0.894 iv. F1 score: 0.902	High accuracy demonstrated by all tested DCNNs Accuracy: Finely tuned VGG16 > Finely tuned VGG19 > VGG16-transfer learning > VGG19 transfer-learning > Basic CNN
Lee and Jong, 2020, Republic of Korea [51]	- DCNN - GoogLeNet Inception v3	- Depth: 22 layers deep, 2 fully connected layers - Training epochs: 1000	PA and Pano	January 2010 to December 2019	*N* = 10,770 (Pano: 5390, PA: 5380)	Implant brands: *N* = 3 (A) Osstem TSIII implant system (42.71%) (B) Dentium Superline implant system (40.57%) (C) Straumann BLT implant system (16.71%)	DCNN vs. board-certified periodontist	Test Group: 20% Training Group: 60% Validation Group: 20%	i. AUC: 1. Overall: - DCNN: 0.971 (95% CI: 0.963–0.978) - Periodontist: 0.925 (95% CI: 0.913–0.935) 2. Pano: - DCNN: 0.956 (95% CI: 0.942–0.967) - Periodontist: 0.891 (95% CI: 0.871–0.909) 3. PA - DCNN: 0.979 (95% CI: 0.969–0.987) - Periodontist: 0.959 (95% CI: 0.945–0.970) ii. Sensitivity and specificity 1. Overall: - DCNN: 95.3% and 97.6% - Periodontist: 88.7% and 87.1% 2. Pano: - DCNN: 93.6% and 95.7% - Periodontist: 82.9% and 90.3% 3. PA - DCNN: 97.1% and 99.5% - Periodontist: 94.2% and 95.8%	DCNN is useful for the identification and classification of DISs Accuracy: DCNN > Periodontist (both are reliable)
Kim et al., 2020, Republic of Korea [55]	5 different CNNs (1) SqueezeNet (2) GoogLeNet (3) ResNet-18 (4) MobileNet-v2 (5) ResNet-50	- Depth: (1) 18 layers (2) 22 layers (3) 18 layers (4) 54 layers (5) 50 layers - Training epochs: 500	PA	2005 to 2019	*N* = 801	Implant models: *N* = 4 1. Brånemark Mk TiUnite 2. Dentium Implantium 3. Straumann Bone Level 4. Straumann Tissue Level	EORS	NM	Test accuracy: (a) SqueezeNet: 96% (b) GoogLeNet: 93% (c) ResNet-18: 98% (d) MobileNet-v2: 97% (e) ResNet-50: 98% Precision: (a) SqueezeNet: 0.96 (b) GoogLeNet: 0.92 (c) ResNet-18: 0.98 (d) MobileNet-v2: 0.96 (e) ResNet-50: 0.98 Recall: (a) SqueezeNet: 0.96 (b) GoogLeNet: 0.94 (c) ResNet-18: 0.98 (d) MobileNet-v2: 0.96 (e) ResNet-50: 0.98 F1 score: (a) SqueezeNet: 0.96 (b) GoogLeNet: 0.93 (c) ResNet-18: 0.98 (d) MobileNet-v2: 0.96 (e) ResNet-50: 0.98	CNNs can classify implant fixtures with high accuracy

DCNN, deep convolutional neural network; CNN, convolutional neural network; PA, periapical radiograph; Pano, panoramic radiograph; NM, not mentioned; DL, deep learning; EORS, expert opinions, reference standards; YOLO, you only look once; NB, Nobel Biocare; ST, Straumann; ZB, Zimmer Biomet; AUC, area under the receiver operating characteristic curve; PPV, positive predictive value; NPV, negative predictive value; VGG, Visual Geometry Group, Oxford University; CI, confidence interval; DISs, dental implant systems; ABN, attention branch network; *, details not mentioned due to high number; FCN, fully convolutional network; IPA, image processing augmentation; R-CNN, region-based convolutional neural network; mAP, mean average precision.

## Data Availability

The data that support the findings of this study are available from the corresponding author upon reasonable request.

## Supplementary Materials

The following supporting information can be downloaded at: https://www.mdpi.com/article/10.3390/diagnostics14080806/s1, Table S1: Quality Assessment (QUADAS-2) Summary of Risk Bias and Applicability Concerns.
