# Supplementary material for: Accuracy of Artificial Intelligence Models in Dental Implant Fixture Identification and Classification from Radiographs: A Systematic Review
Source: Diagnostics (Basel). 2024 Apr 11;14(8):806. doi: 10.3390/diagnostics14080806 (PMC11049199; doi:10.3390/diagnostics14080806)
Supplement: Supplementary file 1 [file diagnostics-14-00806-s001.zip › diagnostics-2910040-supplementary.pdf]

Table S1. Quality Assessment (QUADAS-2) summary of Risk Bias and Applicability concerns

Study	RISK OF BIAS				APPLICABILITY CONCERNS		
	PATIENT SELECTION	INDEX TEST	REFERENCE STANDARD	FLOW AND TIMING	PATIENT SELECTION	INDEX TEST	REFERENCE STANDARD
Kong et al., 2023 [61]	☺	☺	☺	☺	☺	☺	☺
Kong, 2023 [58]	☺	☺	?	☺	☺	☺	?
Park et al., 2023 [62]	☺	☺	☺	☺	☺	☺	☺
Hsiao et al., 2021 [63]	☺	☺	☹	☺	☺	☺	☹
Park et al., 2023 [48]	☺	☺	☺	☺	☺	☺	☺
Kong et al., 2023 [31]	☺	☺	?	☺	☺	☺	?
Jang et al., 2022 [57]	☺	?	☺	☺	☺	?	☺
Kohlakala et al., 2022 [32]	☺	☺	☺	☺	☺	☺	☺
Sukegawa et al., 2022 [30]	☺	☺	☺	☺	☺	☺	☺
Kim et al., 2022 [59]	☺	☺	?	☺	☺	☺	?
Lee et al., 2022 [60]	☺	☺	?	☺	☺	☺	?
Benakatti et al., 2021 [56]	☺	?	☺	☺	☺	?	☺
Santos et al., 2021 [49]	☺	☺	?	☺	☺	☺	?
Sukegawa et al. , 2021 [52]	☺	☺	?	☺	☺	☺	?
Lee et al., 2021 [54]	☺	☹	☺	☺	☺	☹	☺
Hadj Saïd et.al, 2020 [46]	☺	☺	?	☺	☺	☺	?
Lee et al., 2020 [53]	☺	☺	☺	☺	☺	☺	☺
Takahashi et al., 2020 [47]	☺	☺	?	☺	☺	☺	?
Sukegawa et al., 2020 [50]	☺	☺	?	☺	☺	☺	?
Lee & Jong, 2020 [51]	☺	☺	☺	☺	☺	☺	☺
Kim et al., 2020 [55]	☺	?	?	☺	☺	?	?

☺ Low Risk ☹ High Risk ? Unclear Risk
